# A Text-Book of Gynecology

**Published:** 1902-09

**Authors:** 


A Text-Book of Gynecology. Edited by Chas. A. L. Reed, A. M., M. D., of Cincinnati, President of the American Medical Association from 1900 to 1901; Gynecologist and Clinical Lecturer on Surgical Diseases of Women at the Cincinnati Hospital, etc. Illustrated by R. J. Hopkins. Pages, 900. Price, $5.00. New York: D. Appleton & Co. 1901.

This work has the proper size and contents for a typical textbook. The author is not alone in the preparation of the volume. The best talent has contributed to the various subjects, and the third person has been given to all of the subject matter. As a working manual for practitioners and students the present volume has no superior and but few equals. T. J. B.

